# Supplementary material for: Using Electronic Reminders to Improve Human Papillomavirus (HPV) Vaccinations among Primary Care Patients
Source: Vaccines (Basel). 2023 Apr 20;11(4):872. doi: 10.3390/vaccines11040872 (PMC10145812; doi:10.3390/vaccines11040872)
Supplement: Supplementary file 1 [file vaccines-11-00872-s001.zip › vaccines-2339220-supplementary.pdf]

Supplementary Table S1. Baseline Characteristics of secondary analysis

Baseline Characteristics	Electronic Reminder (N=2,768) N (%)	Usual Care (N=3,703) N (%)	P-value
Age			<0.000
9-14	930 (33.60)	1,535 (41.45)	
15-18	576 (20.81)	693 (18.71)	
19-25	1,262 (45.59)	1,475 (39.83)	
Sex			0.357
Male	1,218 (44.00)	1,672 (45.15)	
Female	1,550 (56.00)	2,031 (54.85)	
Vaccine Status			0.240
Not initiated	1,943 (70.20)	2,549 (68.84)	
Initiated	825 (29.80)	1,154 (31.16)	
Race/Ethnicity			0.921
Non-Hispanic White	693 (25.04)	939 (25.36)	
Non-Hispanic Black	706 (25.51)	962 (25.98)	
Hispanic	671 (24.24)	874 (23.60)	
Other/Unknown	698 (25.22)	928 (25.06)	
Insurance			0.009
Medicaid	898 (32.44)	1,337 (36.11)	
Private (managed Care)	1,619 (58.49)	2,049 (55.33)	
Uninsured	221 (7.98)	291 (7.86)	
Other	30 (1.08)	26 (0.70)	

Supplementary Table S2. Secondary analysis* of HPV outcomes

HPV outcomes	Electronic Reminder (N=2,768)			Usual Care (N=3,703)
	N (%)	Adjusted Odds Ratio (95% CI**)	P-Value	N (%)
Appointment Scheduling	563 (20.3)	1.20 (1.06, 1.37)	0.005	700 (18.9)
Clinic Visit	403 (14.56)	1.13 (0.98, 1.31)	0.088	520 (14.04)
All additional HPV Vaccination(s)	333 (12.03)	1.35 (1.14, 1.59)	<0.001	402 (10.86)

*Model controlled for age, sex, race/ethnicity, insurance and vaccine status

Supplementary Table S3. secondary analysis of subgroup vaccination outcomes

	Electronic Reminder (N=1,931)			Usual Care (N=2,538)
	N (%)	Adjusted Odds Ratio (95% CI**)	P-Value	N (%)
HPV vaccine initiation rate	146 (7.56)	1.32 (1.04, 1.68)	0.021	181 (7.13)

	Electronic Reminder (N=2,768)			Usual Care (N=3,703)
	N (%)	Adjusted Odds Ratio (95% CI**)	P-Value	N (%)
HPV vaccine completion rate	168 (6.07)	1.32 (1.12, 1.56)	0.001	207 (5.59)
